# Nutraceuticals and Nutrition Supplements: Challenges and Opportunities

**DOI:** 10.3390/nu12061593

**Published:** 2020-05-29

**Authors:** Rafat A. Siddiqui, Mohammed H. Moghadasian

**Affiliations:** 1 Food Chemistry and Nutrition Science Laboratory, Agricultural Research Station, Virginia State University, Petersburg, VA 23806, USA; 2 Department of Food and Human Nutritional Sciences, University of Manitoba, Winnipeg, MB R3T 2N2, Canada; mmoghadasian@sbrc.ca; 3 Canadian Center for Agri-food Research in Health and Medicine, St. Boniface Hospital Research Center, Winnipeg, MB R2H 2A6, Canada

The term “nutraceuticals” is derived from “nutrition” and “pharmaceuticals” and is used for nutrition products that are also used as medicine [1]. “Nutraceuticals” often contain modified/unmodified whole food, plant extracts alone or in combination, semipurified and purified phytochemicals, or a combination of different phytochemicals. On the other hand, nutritional supplements are nutritional compounds that supplement one’s diet by increasing one’s total daily intake. Nutritional supplements also contain substances alone or in combination with vitamins and minerals, with or without other herbal products, with or without zoochemicals (creatinine, glucosamine, melatonin, bee pollens) and with or without probiotics. 

Nutraceuticals and nutrition supplements are collectively referred to as “dietary supplements,” intended to be taken orally [2]. The use of supplements is suggested to (but may not claim to) diagnose, cure, mitigate, treat, or prevent diseases. Often, background information suggests that they are intended to affect the structure or function of the body [3]. However, they do not undergo premarket approval. The common reasons for using dietary supplements are to improve conditions such as overall health and disease prevention, performance (athletics, sports, sex, etc.) and appearance (weight loss, sex appeal) [4]. These are often perceived as “safe” and less likely to have side effects. The scientific research on nutraceuticals and nutrition supplements is frequently misinterpreted or overstretched for commercial interests because of high consumer demands. The manufacturing and marketing of supplements are full of challenges.

Several challenges associated with the development of nutraceuticals are often ignored because of a lack of authoritative control. These challenges include identification of the authentic source of raw materials, purity of the compound, presence of other active compounds, quality, lack of experimental evidence, false advertising, contamination with heavy metals, and interactions between supplements and drugs. For example, a common herb “ginseng” has several varieties [5] such as California ginseng, wild ginseng, prickly ginseng, Pacific ginseng, Malaysian ginseng, Indian ginseng, Peruvian ginseng, Southern ginseng, Brazilian ginseng, and wild-red ginseng. All of these are sold as ginseng, but none of these belongs to the genus *Panax*, which contains real ginseng including Korean ginseng (*P. ginseng*), South China ginseng (*P. notoginseng*) and American ginseng (*P. quinquefolius*). Some varieties of star anise have several hundred-fold anisatin, a neurotoxin, that the authentic star anise (*Illicium verum*) has [6]. The supplements that are not prepared under strict GMP conditions may have unintentional contamination [7], including microbes (pathogens/nonpathogens), pesticides, mycotoxin (aflatoxin), heavy metals (seaweeds), zinc (cadmium), and calcium (lead). In addition, some supplements such as those commonly used for weight loss, body building, and sex enhancement are spiked with prohibited drugs to improve efficacy [8]. Some manufacturers also try to use a closely related herb, which may or may not have the active ingredients. For example, goldenseal (*Hydrastis canadensis*), used for berberine/hydrastine content, is often substituted with goldthread (*Coptis chinensis*) or Oregon grape (*Mahonia aquifolium*), which may have low or no berberine/hydrastine [9]. 

Besides, maintaining the quality of nutraceuticals is another challenge, as phytochemistry is inherently variable due to seasonal and geographical variations [10]. It is challenging to measure and maintain consistency in finished products and limit undesirable constituents. Interaction of herbal supplements with medicinal drugs is also a big concern [11]. For example, St. John’s wort (*Hypericum perforaum*) is used as an effective antidepressant, but it also activates several cytochrome P450 isoenzymes, which make a large number of medicinal drugs ineffective [12,13]. Among all the major concerns for using the nutraceuticals is the lack of scientific evidence [14]. Some are never tested under properly controlled experimental conditions, and unlike pharmaceuticals, most nutraceuticals do not undergo “randomized controlled clinical studies.” 

The purpose of this Special Issue on “Nutraceuticals, Nutrition Supplements, and Human Health” is to comprehensively review the data from basic and clinical research to discuss the benefits as well as potential adverse effects of “functional food-derived” products. We have invited international experts, researchers and authors to submit original research and review articles that address the progress and our current understanding of nutraceuticals/supplements from in vitro and in vivo studies, as well as from clinical trials describing the benefits/adverse effects with underlying mechanisms. This Special Issue presents a compendium of excellent laboratory and clinical studies using plant extracts [15,16,17,18,19,20], purified compounds [21,22,23], modified formulations [22,24], and probiotics [25,26,27,28] to improve many health conditions, including metabolic disorders [17,20], cardiovascular disease [29], muscle metabolism [18,25], obesity [16,17,20], neurological disorders [30], infectious diseases [26,27], aging [23], and cancers [18,21,24,31].

This Special Issue’s overall goal is to present readers with high-quality scientific evidence for the use of dietary supplements, nutraceuticals, and functional foods that can be appropriately used to improve health parameters in various stages of one’s lifecycle. We thank all the contributors for their contributions and for their dedication to making a difference in human health with low-cost remedies.
